# Is the Clinical Version of the Iowa Gambling Task Relevant for Assessing Choice Behavior in Cases of Internet Addiction?

**DOI:** 10.3389/fpsyt.2019.00232

**Published:** 2019-05-27

**Authors:** Ching-Hung Lin, Chao-Chih Wang, Jia-Huang Sun, Chih-Hung Ko, Yao-Chu Chiu

**Affiliations:** ^1^Department of Psychology, Kaohsiung Medical University, Kaohsiung, Taiwan; ^2^Research Center for Nonlinear Analysis and Optimization, Kaohsiung Medical University, Kaohsiung, Taiwan; ^3^Department of Psychology, Soochow University, Taipei, Taiwan; ^4^Research Center for Education and Mind Sciences, National Tsing Hua University, Hsinchu, Taiwan; ^5^Department of Psychiatry, Kaohsiung Medical University Hospital, Kaohsiung, Taiwan; ^6^Graduate Institute of Medicine, College of Medicine, Kaohsiung Medical University, Kaohsiung, Taiwan

**Keywords:** Internet addiction (IA), Internet gaming disorder (IGD), Iowa gambling task (IGT), expected value, gain–loss frequency, prominent deck B phenomenon, decision-making

## Abstract

**Objective:** A critical issue in research related to the Iowa gambling task (IGT) is the use of the alternative factors expected value and gain–loss frequency to distinguish between clinical cases and control groups. When the IGT has been used to examine cases of Internet addiction (IA), the literature reveals inconsistencies in the results. However, few studies have utilized the clinical version of IGT (cIGT) to examine IA cases. The present study aims to resolve previous inconsistencies and to examine the validity of the cIGT by comparing performances of controls with cases of Internet gaming disorder (IGD), a subtype of IA defined by the fifth edition of the *Diagnostic and Statistical Manual of Mental Disorders*.

**Methods:** The study recruited 23 participants with clinically diagnosed IGD and 38 age-matched control participants. Based on the basic assumptions of IGT and the gain–loss frequency viewpoint, a dependent variables analysis was carried out.

**Results:** The results showed no statistical difference between the two groups in most performance indices and therefore support the findings of most IGT-IA studies; in particular, expected value and gain–loss frequency did not distinguish between the IGD cases and controls. However, the participants in both groups were influenced by the gain–loss frequency, revealing the existence of the prominent deck B phenomenon.

**Conclusion:** The findings provide two possible interpretations. The first is that choice behavior deficits do not constitute a characteristic feature of individuals who have been diagnosed with IGD/IA. The second is that, as the cIGT was unable to distinguish the choice behavior of the IGD/IA participants from that of controls, the cIGT may not be relevant for assessing IGD based on the indices provided by the expected value and gain–loss frequency perspectives in the standard administration of IGT.

## Background

### Internet Gaming Disorder

In the past decade, with the rising popularity of the Internet, Internet addiction (IA) has been used as a global term to describe all types of Internet compulsion and dependence, such as Internet gaming disorder (IGD), communication addiction disorder, and virtual reality addiction. However, defining these subtypes has been a controversial issue. For instance, an increasing number of people are devoted to international online gaming competitions and undergo related training sessions for about 6 h a day; however, these players reject the label of “Internet gaming disorder” and would rather their activities be labeled as “online gaming athletics.” The definitions of IA and IGD as well as complete clinical models are still under construction and validation (1).

IA and IGD have become frequently discussed clinical issues (2, 3). In May 2013, IGD became a newly defined psychiatric symptom as a condition necessitating further research in the appendix to the fifth edition of the American Psychiatric Association’s *Diagnostic and Statistical Manual of Mental Disorders*, 5th edition (DSM-5) (4, 5). Notably, Young (2, 3) first identified these Internet-related behavioral malfunctions and enrolled the clinical definition (in DSM-IV) of pathological gambling and substance addiction to describe them.

In fact, the criteria for IGD were used to define Internet use disorder in DSM-5, which demonstrated the similarity in symptoms between the two disorders. However, epidemiological study has demonstrated their differences in associated factors, such as gender or self-esteem (6). Thus, whether IGD is a type of IA or a type of gaming disorder, or a type of both, is still a subject of debate. However, only IGD was recruited to describe Internet use disorder in DSM-5. The increasing indication of clinically remarkable harm derived from excessive game playing, such as death, deep vein thrombosis (7), or seizure, suggests that this is a noteworthy situation from a public health perspective (4).

IA-related research began attempts to measure IA-related psychological traits, mental state, and behavioral patterns, by developing IA-related assessment tools and questionnaires, such as Young’s Internet Addiction Scale (YIAS) (2, 3) and Chen’s Internet Addiction Scale (CIAS) (8). However, there were difficulties in categorizing IA as a single neuropsychiatric condition as it was found to involve very broad behavioral problems whose symptoms may converge with other neuropsychiatric conditions (9, 10). Furthermore, few game-based assessment tools had been developed for evaluating the choice behavior of IA or IGD cases. Consequently, prompted by the fast development of technology and the Internet, researchers have tried varied decision tasks to measure IGD- or IA-related behavior due to its similarity with the symptoms defined by pathological gambling and substance addiction. The Iowa gambling task (IGT) was found to be one of the most ecological tools to evaluate the choice behavior of IA cases.

### The Iowa Gambling Task

The IGT is a dynamic decision task developed by Bechara et al. (11), following research in neuroscience. Damasio (12) proposed an emotion-decision theory called the Somatic Marker Hypothesis to interpret the real-life decision-making problems of patients suffering from lesions of the ventromedial prefrontal cortex (VMPFC). It was found that VMPFC patients possessed normal IQ scores and normative responses in facing some moral questions, but had problems with decision-making under uncertainty (12, 13). The IGT was designed to quantitatively measure the decision patterns and dysfunction of VMPFC patients under these conditions of uncertainty. There are now over 900 articles that have utilized the IGT as a research tool to assess choice behavior under conditions of uncertainty. Most of the IGT serial studies showed that the choice patterns of some neurological and psychiatric deficits with decision dysfunction can be successfully distinguished from those of healthy controls; the psychiatric deficits have included amygdala lesions, substance addiction, and pathological gambling (14–18). Importantly, the IGT has been considered as a critical and relatively ecological tool in assessing the choice behavior that is modulated by the emotive system in an uncertainty situation, including implicit and explicit processing. Consequently, an increasing number of behavioral addiction studies have enrolled the IGT as an assessment tool to evaluate the decision dysfunction (18) in such cases, including IGD (19–28). A series of neuropsychiatric studies have suggested that the IGT is able to distinguish the choice patterns of controls from those of substance addiction cases (15–17) as well as pathological gambling cases (18).

There are four decks in the IGT and each deck has a very different gain–loss structure. Decks A and B enable decision-makers to gain $100 in each trial, but in some trials deck A makes them lose $150 to $350 while deck B makes them lose $1,250. The two decks are called bad decks due to the negative outcome (a loss of $250) on an average of 10 trials. Conversely, deck C enables decision-makers to gain $50 in each trial, but in some trials deck C makes them lose $25 to $75 while deck D makes them lose $250. The two decks are called good decks due to the positive outcome (a gain of $250) on an average of 10 trials (11). Remarkably, in the original IGT sequence (consisting of four cycles of 10 trials), the gain–loss frequency of decks A and C as well as that of decks B and D are ideally counterbalanced. **Table 1** details the long-term outcome and gain–loss sequence of each deck. The participants in Bechara et al.’s (11) version were informed that they should aim to win money if they possibly can, or avoid losing money if they possibly can. Further, the participants had no knowledge of the gain–loss structure and the end point of the game (14, 29).

**Table 1:** The first cycle of 10 trials in the gain–loss structure of IGT.

Deck	A		B		C		D	
Trial	Gain	Loss	Gain	Loss	Gain	Loss	Gain	Loss
1	100	0	100	0	50	0	50	0
2	100	0	100	0	50	0	50	0
3	100	−150	100	0	50	−50	50	0
4	100	0	100	0	50	0	50	0
5	100	−300	100	0	50	−50	50	0
6	100	0	100	0	50	0	50	0
7	100	−200	100	0	50	−50	50	0
8	100	0	100	0	50	0	50	0
9	100	−250	100	−1,250	50	−50	50	0
10	100	−350	100	0	50	−50	50	−250
Net value	$−250		$−250		$+250		$+250	
Gain–loss frequency	10 gains 5 losses		10 gains 1 loss		10 gains 5 losses		10 gains 1 loss	

Since 2007, the IGT has been developed as a standard clinical evaluation tool for assessing a range of psychological disorders, as mentioned above. The clinical version of IGT (cIGT) (30) possesses most of the variables in the gain–loss structure of the original IGT (11), with revisions to only a few components. For instance, the gain–loss sequence of cIGT was extended from four cycles (40 trials) to six cycles (60 trials). Additionally, the value contrast between good decks (C, D) and bad decks (A, B) gradually increases cycle-by-cycle. Specifically, the long-term outcome of decks A and B in cIGT becomes increasingly negative in consecutive cycles compared to the outcome in the original version of IGT, whereas the long-term outcome of decks C and D in cIGT becomes increasingly positive in consecutive cycles compared to the original version (for a detailed comparison of the two versions of IGT, please see **Supplementary Table S1**) (31). Additionally, the second version of the clinical IGT, published in 2016 (32), extended the age range of the norm.

### The New Interpretative Factor: Gain–Loss Frequency

Claims for the relevance of the IGT in assessing disorders now cover 13 clinical neuropsychiatric disorders or syndromes, including affective disorders. Nevertheless, over the past two decades, numerous IGT studies have pointed out that not only neuropsychiatric patients but also even healthy decision-makers exhibit myopic decision patterns in the IGT (33–48). Specifically, most healthy decision-makers prefer to choose bad deck B more than bad deck A, and in numbers almost equal to good decks C and D. This shortsighted choice behavior is the so-called “prominent deck B phenomenon” (34, 36, 37), which demonstrated that the selection behavior of decision-makers in the IGT was mostly dominated by gain–loss frequency rather than by the expected value. That is to say, decision-makers prefer to choose the option with a frequent gain and avoid the options with frequent losses, without taking into account the final outcome of good expected value decks C and D (49).

In the past, most IGT-related studies have utilized the expected value score [(C+D)–(A+B)] to present their data and have not considered the prominent deck B phenomenon and the gain–loss frequency effect (33, 34, 36, 37, 46). In recent years, however, an increasing amount of IGT-related studies have calculated the mean numbers of each deck to present and analyze their data, and have argued that more attention should be paid to the prominent deck B phenomenon (33, 34, 36, 37, 42, 46). Notably, some IGT studies have enrolled the prominent deck B phenomenon as a behavioral index to evaluate decision-making behavior (40, 41, 43, 45). In fact, most of the studies using the four-deck presentation have demonstrated that gain–loss frequency overrode expected value in guiding decision-making behavior under conditions of uncertainty (42). For example, Upton et al. showed that gain–loss frequency is relatively more powerful than expected value in distinguishing the choice patterns of opiate users from those of controls (45). Seeley et al. utilized gain–loss frequency to assess decision-making behavior in their serial sleep deprivation studies (40, 41). In IGT-modeling studies, Ahn et al. and Worthy et al. demonstrated that models related to gain–loss frequency are relatively more influential than models based on expectancy-learning theories (48, 50, 51). However, it is worth noting that the special issue of *Frontiers in Psychology* entitled “Twenty Years After the Iowa Gambling Task: Rationality, Emotion, and Decision-Making” lists three critical issues (33) that need further clarification, as follows: 1) the issue of implicit vs. explicit processes in analyzing influences on decision-making behavior (52, 53); 2) the issue of the representation of skin conductance response (15, 54); and 3) the issue of gain–loss frequency vs. expected value in analyzing the relative power of their influence on decision-making behavior (33).

### Inconsistencies in IGT-IA Related Studies

Based on the similarities in behavioral symptoms and pathological mechanisms between IA (2, 3) and both pathological gambling and substance addiction, several research groups have enrolled the IGT to compare the choice behavior of IA cases, including IGD, with that of controls. The present research reviewed 10 IGT-IA studies and found that the results were inconsistent (**Table 2**). For example, some studies suggest that control groups choose more advantageous decks than IA cases (19–21). Sun et al. (20) suggest that excessive Internet users perform poorly in the IGT compared to a control group. In two separate studies, Xu (21) and Zhang (19) show that IA and IGD cases perform poorly in the IGT compared to healthy controls. Conversely, other studies suggest that IA cases perform better than controls in the IGT (22, 28). Zheng (22) revealed that the IGT performance of IA cases was better than healthy controls. Ko et al. (9) demonstrated that IA cases perform better than non-addicted Internet users in the last stages (40 trials) of the IGT. Moreover, there are some IGT-IA studies that have demonstrated a lack of difference in the IGT performances between IA cases and controls (23–27). Liang and You (23) found no significant differences in IGT performance between IA cases and healthy controls. Song et al. (26) used the four-deck format to detail IGT performance and found no significant difference between IA cases and non-addicted Internet users. Metcalf and Pammer (24) and Yao et al. (27) compared the IGT performance of IGD cases with non-IGD cases and found no significant difference between the two groups. Nikolaidou et al. (25) also found no significant difference in IGT performance between problematic Internet users and nonproblematic Internet users.

**Table 2:** The results of net score comparisons in the IGT-IA/IGD literature.

Studies	Participants	Index	Results
Sun et al. (20)	EIU (42M, 10F); CON (26M, 16F)	[(C+D)-(A+B)]	EIU < CON
Xu (21) (in Chinese)	IA (32M, 10F); HC (26M, 16F)	5 blocks [(C+D)-(A+B)]	IA < HC
Zhang (19) (in Chinese)	IGD (30M, 6F); HC (30M, 6F)	5 blocks [(C+D)-(A+B)]	IGD < HC
Zheng (22) (in Chinese)	IA (22); HC (21)	5 blocks [(C+D)-(A+B)]	IA > HC
Ko et al. (9)	IA (53M, 21F); InA (56M, 58F)	Last 40 cards [(C+D)-(A+B)]	IA > InA
Liang and You (23) (in Chinese)	IA (18M, 4F); HC (18M, 4F)	[(C+D)-(A+B)]	IA = HC
Song et al. (26) (in Chinese)	IA (54); InA (151)	4 decks [A,B,C,D]	IA = InA
Metcalf and Pammer (24)	HE (25M); NG (22M)	[(C+D)-(A+B)]	A = NG
Yao et al. (27)	IGD (34); HC (32)	All trials [(C+D)-(A+B)]	IGD = HC
Nikolaidou et al. (25)	PIU (27); NPIU (45)	5 blocks [(C+D)-(A+B)]	PIU = NPIU

IA, Internet addiction; InA, Internet non-addiction; IGD, Internet gaming disorder; A, addicted; NG, non-gamers; PIU, problematic Internet users; NPIU, nonproblematic Internet users; EIU, excessive Internet users; CON, control.

In summary, these IGT-IA studies arrive at very different conclusions in distinguishing the choices of IA cases from those of controls. It is therefore important to resolve these differences before utilizing the IGT as an assessment tool for IA evaluation. Furthermore, most of the 10 studies utilized the original version of IGT (11) to test the IA and control groups, not the cIGT (30, 32). It would therefore be valuable to resolve these issues using the cIGT. Furthermore, only 2 of these 10 studies (21, 26) took note of the newly considered effects of gain–loss frequency in the IGT. The two studies both provide some discussion of the influence of gain–loss frequency in the IGT, but reach different conclusions based on the expected value and gain–loss frequency viewpoints (21, 26).

### The Aims of the Current Study

The present study aims firstly to test whether the cIGT is a valid tool to distinguish between the choice patterns of IGD cases and those of controls. The second aim is to resolve the issue of inconsistency among the 10 studies mentioned above (19–28). Additionally, we launch here a method of detailed analysis to depict the choice patterns of the two groups. This involves the four-deck format; expected value score [(C+D)–(A+B)]; gain–loss frequency score [(B+D)–(A+C)]; and the learning curves for each deck, using five blocks of 20 trials (i.e., 100 trials in total). Based on the basic expected value assumption of IGT, we propose the following hypotheses. 1) Basically, if the participants prefer the good expected value decks C and D and avoid the bad expected value decks A and B, then the results indicate that the participants will be mostly guided by the expected value in the cIGT. 2) On the other hand, if the participants prefer the high-frequency gain decks B and D and avoid the low-frequency gain decks A and C, then the results indicate that the participants’ decision-making behaviors will be mostly dominated by the prospect of immediate gain. 3) If the cIGT is capable of distinguishing between the dynamic decision abilities of IGD cases and those of controls, we will observe statistically significant differences between the two groups in the IGT indices (such as the scores for expected value and gain–loss frequency) and *vice versa*. 4) Furthermore, if the prominent deck B phenomenon is observed in the cIGT in both groups, we hypothesize that the number of deck B selections may be able to distinguish between the decision patterns of the two groups. Clarification of these hypotheses could be critical for resolving the controversial issues in IGT-IA related studies and in verifying the clinical validity of IGT (37) for assessing the IA or IGD population.

## Method

### Participants

Forty-four participants were initially enrolled in this study, but 18 participants were found to be in remission as of the final stage of the assessment, while the datasets for two participants were mislabeled and the dataset for one participant was lost during the data collection process. Therefore, as of the final stage, the study included only 23 participants who were identified as having an IGD by a qualified psychiatrist based on the diagnostic criteria for IGD in the DSM-5 (5). The control group consisted of 38 age-matched participants who were enrolled following the same diagnostic procedure as the IGD group. The demographic data are listed in **Table 3**. Each participant provided written informed consent before participating in this experiment, and the study followed the guidelines of the Helsinki Declaration. The experimental process was approved by the Institutional Review Board of Kaohsiung Medical University Hospital (KMUH; IRB No. 990380).

**Table 3:** The demographic data of both groups.

Group	IA	HC
Number of participants	23	38
Gender (female/male)	4:19	12:26
Mean age (SD)	25.39 (2.04)	25.66 (2.22)

### Materials

The tool utilized in this study was the standardized cIGT, which was published for use in clinical assessments by Bechara *via* PAR Inc. (30). The gain–loss structure of the cIGT is shown in **Supplementary Table S1** [for additional information on the cIGT, please refer to Takano et al. (31)]. Using the table, it is easy to compare the original gain–loss structure of the IGT with that of the clinical version (**Supplementary Table S1**). Meanwhile, it should be noted that the instructions for the two versions of the IGT are almost the same. In brief, participants are instructed to play a four-card computer game. Initially, the participants have no knowledge of the internal rules of the IGT or the duration of the game; rather, they are simply instructed to earn as much money as they can or to avoid losing money to the extent that they can (14, 29).

### Procedure

The participants in both groups were enrolled through advertisements posted on campus bulletin board and the Internet. To assess the participants, a qualified psychiatrist used the criteria of the DSM-5 (5) and the Chen Internet Addiction Scale (CIAS) (8, 28). In addition to these assessments, the participants were given several decision tasks, including a multiresource interference task (55), a computerized questionnaire for economic decision-making based on the Prisoner’s Dilemma (56), the affective Go/No-go task (57), the cups task (58) and the Soochow gambling task (SGT, following by the cIGT) (59). The participants were also measured for body mass index and also given a blood test before or after performing the IGT (this was for the purpose of other research unconnected to the present study). All of the decision tasks (except SGT) and tests were carried out in no particular order. As the present study focused on exploring IGT-related issues, only the clinical IGT performance data were analyzed for the study. Following the interviews and diagnostics, the participants were invited to perform the cIGT.

### Analytical Methods

A repeated-measures ANOVA was carried out to test the expected value, gain–loss frequency, and deck effects, according to the number of cards selection by each participant in each group. To provide a detailed comparison, the study analyzed the expected value score [(C+D) − (A+B)], gain–loss frequency score [(B+D) − (A+C)], and single deck indices of the four decks, with a repeated-measures ANOVA applied to five blocks in each group and a between-group comparison (IGD vs. controls) performed based on the aforementioned indices.

## Results

**Figure 1:**
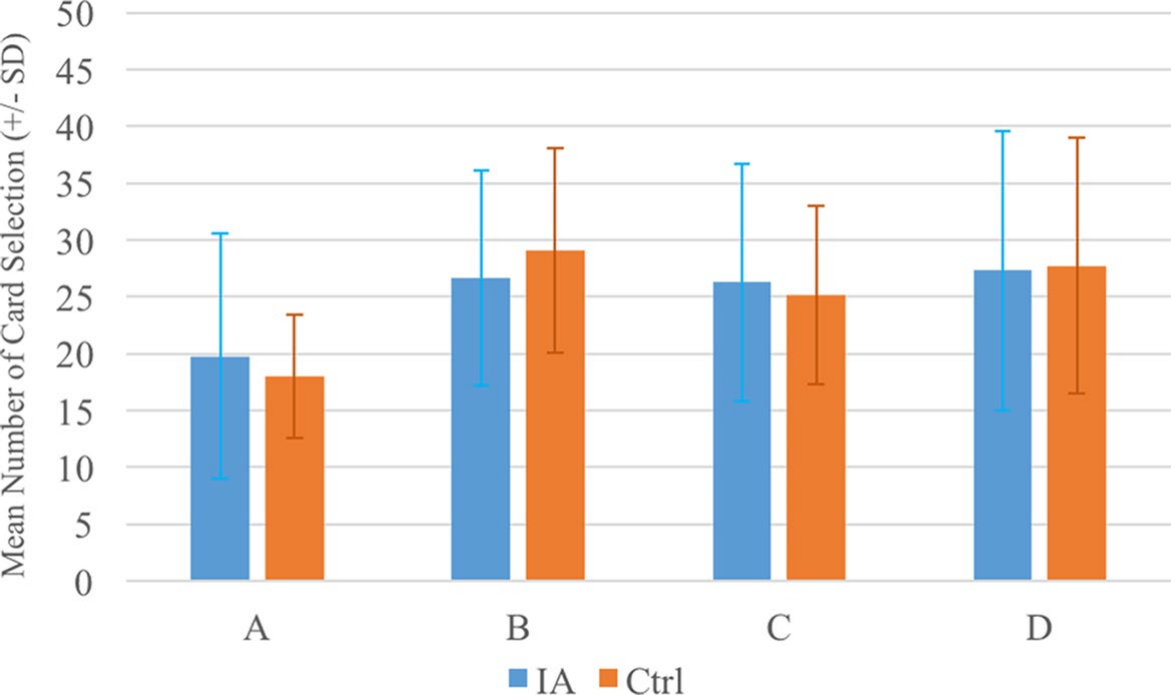
The mean numbers of cards chosen by the two groups. The two groups exhibited similar choice patterns in each deck (see **Table 4**). Notably, the card selection patterns in both groups demonstrated that, in general, decks B, C, and D were preferred rather than deck A, confirming the presence of the prominent deck B phenomenon.

The two-way repeated measures ANOVA of the expected value and gain–loss frequency indices for most conditions in each group showed a lack of significance, but the effects of the gain–loss frequency and the interaction between the expected value and gain–loss frequency were statistically significant in healthy control group (**Table 4** and **Figure 1**).

**Table 4:** The repeated-measurement analysis of expected value and gain–loss frequency indices and interaction effect.

Group	IGD				HC			
	*F*	*df*	*P*	*Eta^2^*	*F*	*df*	*P*	*Eta^2^*
**EV [(C+D)-(A+B)] **	1.29	1, 22	0.27	0.06	2.99	1, 37	0.09	0.08
**GLF[(B+D)-(A+C)]**	3.74	1, 22	0.07	0.15	20.33	1, 37	0.00**	0.36
EV * GLF	1.35	1, 22	0.26	0.06	6.89	1, 37	0.01*	0.16

*p < .05; **p < .01. The repeated-measurement ANOVA was launched to analyze the two factors expected value (EV) and gain–loss frequency (GLF) in each group.

In the IGD group, the repeated-measures ANOVA demonstrated that the deck effect was nonsignificant [*F*(3, 66) = 1.82, *p* = 0.15, *Eta* *^2^* = 0.08]. A significant effect was only observed in comparing decks A and B. In the control group, however, the main effect of deck was statistically significant [*F*(3, 111) = 9.42, *p* < 0.01, *Eta* *^2^* = 0.20], while *post hoc* analysis (least significant difference) revealed statistical significance for the pairs of decks A–B, A–C, and A–D. Details of the statistics are shown in **Figure 1** and **Table 5**.

**Table 5:** The repeated-measurement analysis of deck effect and extended *post hoc* analysis of each group.

Group	IGD			HC		
*Post hoc Analysis*	*Mean Difference*	*s.e.m.*	*P*	*Mean Difference*	*s.e.m.*	*P*
**A–B**	−6.87	2.84	0.02*	−11.13	1.71	0.00**
**A–C**	−6.48	3.92	0.11	−7.21	1.58	0.00**
**A–D**	−7.52	4.13	0.08	−9.76	2.35	0.00**
**B–C**	0.39	3.31	0.91	3.92	2.19	0.08
**B–D**	−0.65	4.10	0.88	1.37	2.93	0.64
**C–D**	−1.04	3.59	0.77	−2.55	2.65	0.34

*p < .05; **p < .01. The single factor (deck: A, B, C, D) repeated-measurement ANOVA was launched here to detail the deck effect and deck-by-deck comparison in post hoc analysis.

**Figure 2:**
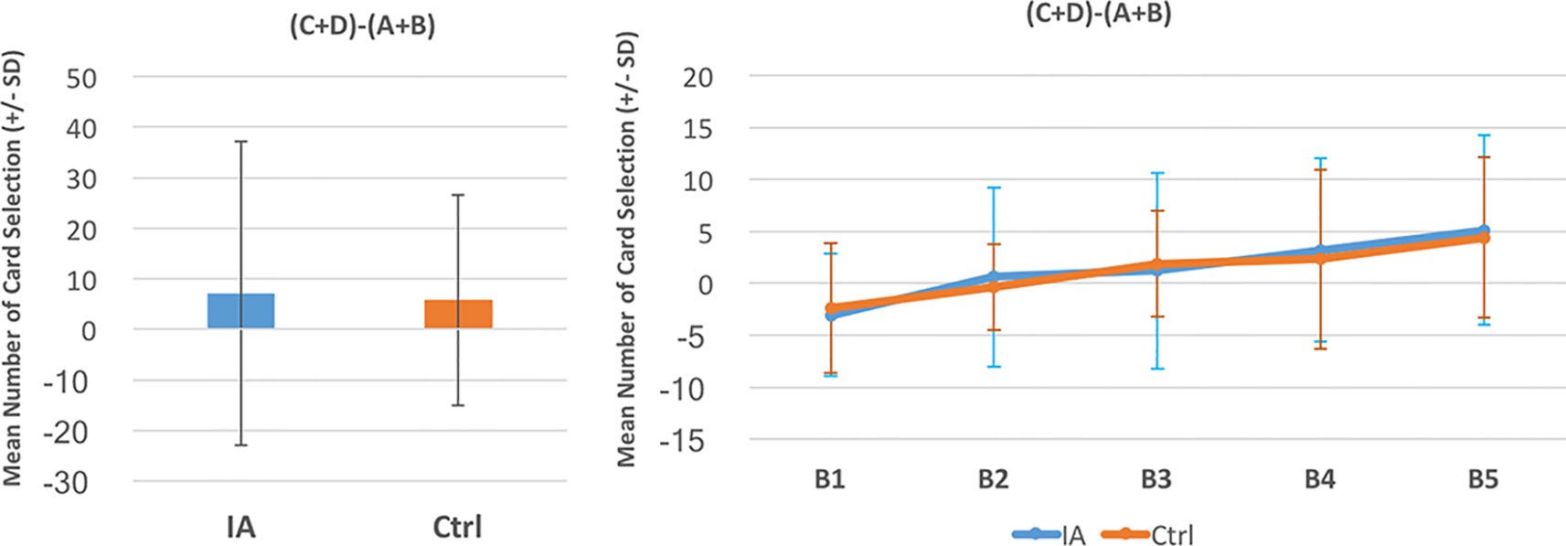
Between-group comparison of the expected value learning curve. Based on the basic assumption of Iowa gambling task (IGT) and expected value, there was no significant difference between the two groups (see **Table 4**). Additionally, the learning curves of both groups revealed the ascending tendency. The present finding, utilizing the cIGT, supports the observations of other studies of this issue (41–45).

**Figure 3:**
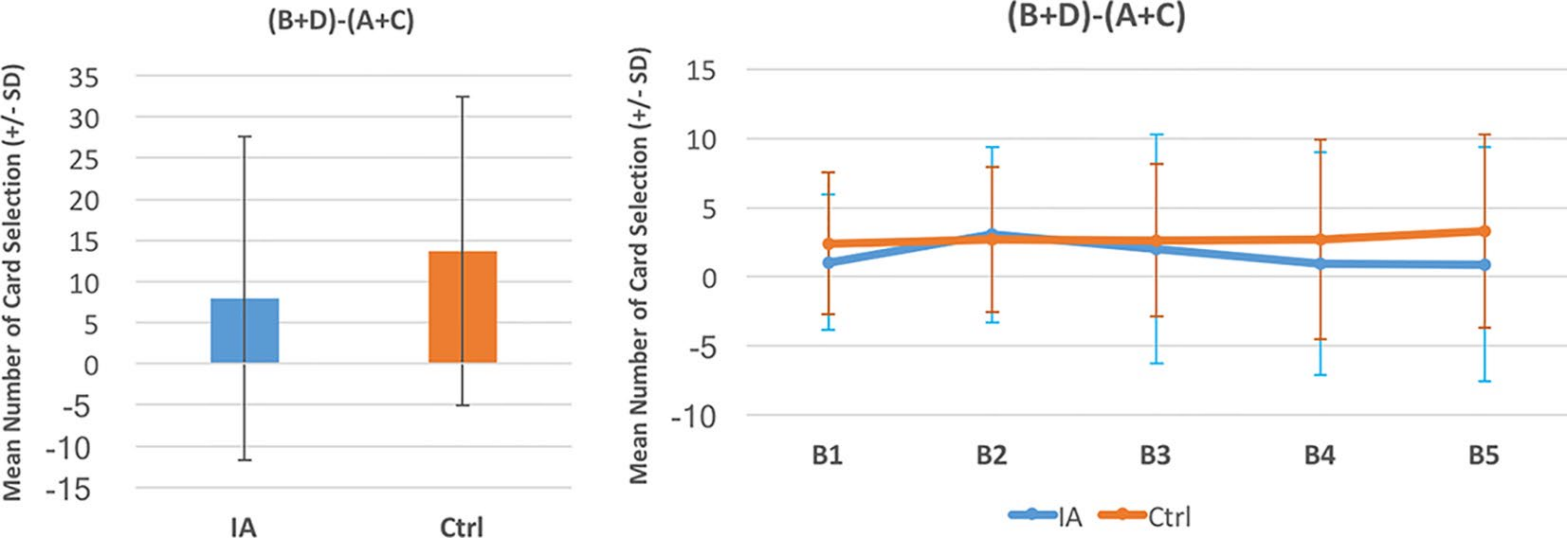
Between-group comparison of the gain–loss frequency learning curve. Based on the gain–loss frequency factor in IGT studies (32), there was no statistical difference between the two groups (see **Table 4**). The learning curves of the gain–loss frequency index in both groups were almost flat from block 1 to block 5. The gain–loss frequency factor has been discussed in several IGT studies (26, 27, 31), but the present analysis did not find evidence for the distinguishable ability of expected value in the standard administration of clinical IGT.

**Figure 4:**
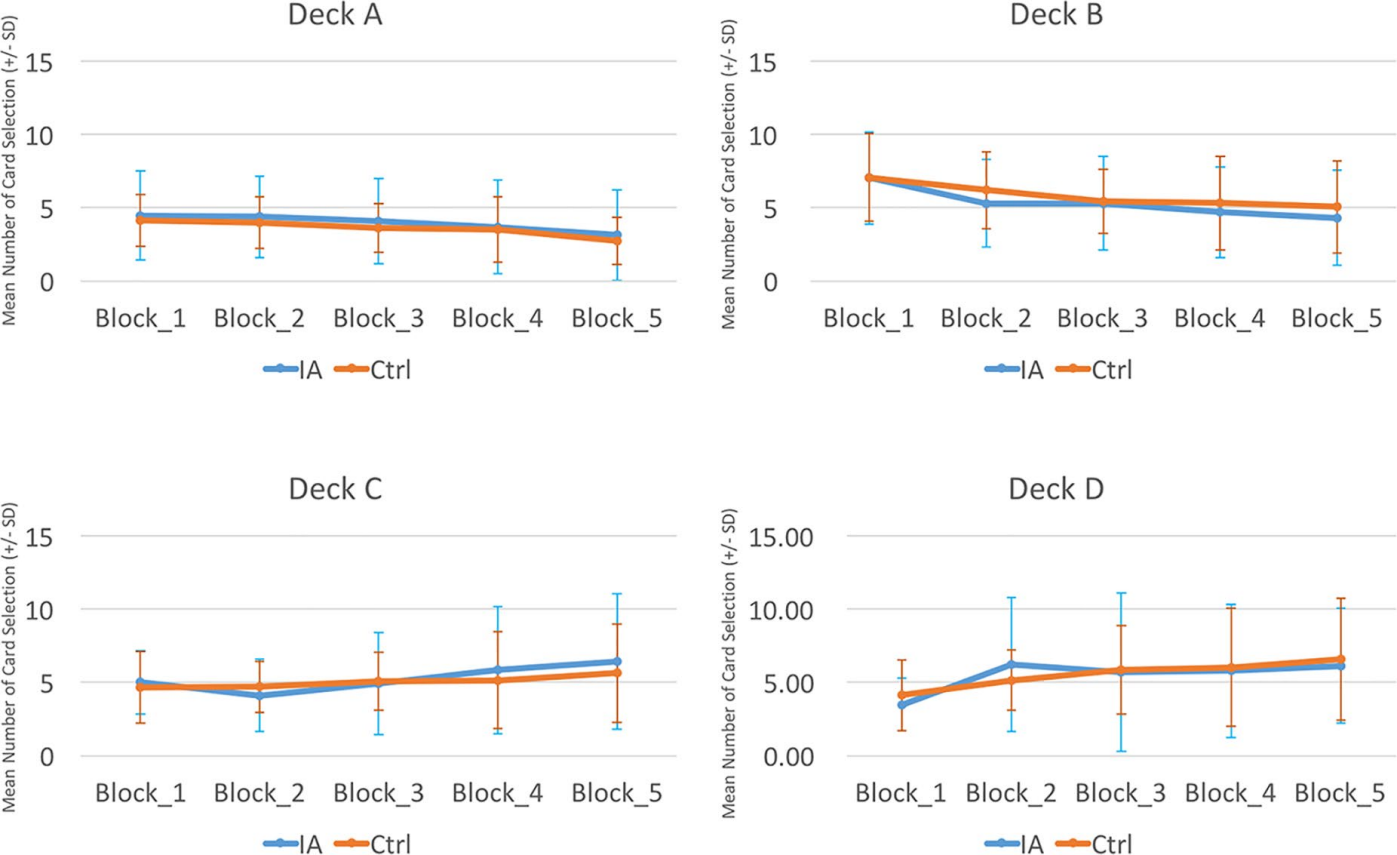
Between-group comparison of learning curves for each deck. The comparison of the two groups demonstrated that differences in the learning curves for each deck were not statistically significant (see **Table 4**). However, it is worth noting that the learning curves of the bad decks (A and B) were slightly descending and those of the good decks (C and D) were slightly ascending. This result suggests that a learning effect does exist, but the standard administration of IGT (100 trials) is too short to reveal the complete learning effect (23).

A between-group comparison of two factors [Group (IGD vs. control) × Blocks (1–5)] was carried out based on each deck and the expected value and gain–loss frequency indices. The repeated-measures ANOVA showed a lack of significance in the group effect, in the interaction of the two factors (Group and Block) with the expected value index [(C+D) − (A+B)] (**Figure 2**) and gain–loss frequency index [(B+D) − (A+C)] (**Figure 3**), and in each deck (**Figure 4** and **Table 6**). However, the block effects in most indices (expected value, A, B, C, D) were significant, with gain–loss frequency being the exception (**Figures 2**, **3**, and **4** and **Table 6**). In sum, the IGD group preferred the four decks in relatively equal measure compared to the control group, but all the results of the between-groups analysis were nonsignificant. Notably, however, the learning (block) effect can be observed in the expected value indices and each single deck. Moreover, most of the participants in both groups preferred deck B rather than deck A, showing the presence of the prominent deck B phenomenon in the current study (**Figure 1**). Additionally, the comparison of the final-net-winning between the IGD and control groups also revealed no significant difference [*t(59)* = −.092, *p* = .927].

**Table 6:** The statistical test of two factors (group and block) repeated measurement in the two indices and each deck.

Variables	Index	*F*	*df*	*P*	*Eta^2^*
**Group**	EV (C+D)-(A+B)	0.04	1	0.84	0.01
	GLF (B+D)-(A+C)	1.49	1	0.23	0.03
	**A**	0.76	1	0.39	0.01
	**B**	1.03	1	0.32	0.02
	**C**	0.21	1	0.65	0.00
	**D**	0.02	1	0.89	0.00
**Block**	EV (C+D)-(A+B)	12.40	4	0.00**	0.17
	GLF (B+D)-(A+C)	0.33	4	0.86	0.01
	**A**	4.57	4	0.00**	0.07
	**B**	7.23	4	0.00**	0.11
	**C**	3.07	4	0.02*	0.05
	**D**	5.65	4	0.00**	0.09
Group * Block	EV (C+D)-(A+B)	0.27	4	0.90	0.01
	GLF (B+D)-(A+C)	0.45	4	0.77	0.01
	**A**	0.05	4	1.00	0.00
	**B**	0.31	4	0.87	0.01
	**C**	0.67	4	0.61	0.01
	**D**	0.68	4	0.61	0.01

*p < .05; **p < .01. The repeated-measurement ANOVA was launched for the group effect (between-group comparison) and the block effect, with five stages of 20 trials on these six indices: expected value (EV), gain–loss frequency (GLF), and decks A, B, C, and D.

## Discussion

### Summary

Over the past two decades, IA has been a controversial issue in clinical psychology and neuropsychiatric medicine. Young (2) adopted the clinical definition of substance addiction and pathological gambling as the preliminary assessment criterion for IA, and some descriptive assessment tools such as CIAS (8) have also been developed. However, due to the disadvantages of self-report questionnaires, these descriptive assessment tools seem incapable of defining some behavioral features of IA in dynamic situations (8). In recent years, therefore, many IA studies have adopted the dynamic and highly uncertain IGT to help in assessing the dynamic decision patterns in IA cases, based on the expected value assumption of the IGT. However, the literature review in the current study has highlighted the contradictory findings of these IGT-IA studies (**Table 2**), with some studies finding that control groups choose more advantageous decks than IA cases (19–21), others finding that the reverse is the case (22, 28), and some detecting no difference between IA cases and controls (23–27).

Using the cIGT, the present study did not find statistically significant differences between an IGD group and a control group. This result supports the findings of other studies (23–27) that the IGT is unable to distinguish between the choice patterns of IA cases and those of controls. There was no statistical difference between the two groups in most IGT indices {decks: A, B, C, D; expected value score [(C+D) − (A+B)]; and gain–loss frequency score [(B+D) − (A+C)]}. The nonsignificance of these scores suggests that even the cIGT may be not relevant for evaluating IGD-related cases due to its limited validity (33, 34, 37, 45). Some learning effects can be observed in the learning curves based on the expected value scores [(C+D) − (A+B)] (**Figure 2**). Nevertheless, the learning effect in the standard running of IGT (100 trials) was incapable of distinguishing between the choice patterns of the two groups. It is worth noting that the prominent deck B phenomenon was revealed in both groups (**Figure 1**). That is, not only the IGD group but also the control group preferred to select bad deck B rather than bad deck A. This finding replicates recent IGT studies, which suggest that the newly revealed factor of the gain–loss frequency may be the main guiding element under dynamic uncertainty situations. This observation is congruent with the gain–loss frequency perspective in recent IGT-related studies (33–48).

### The Basic Assumption of IGT: Expected Value

In the past decade, the IGT has been claimed to be a valid clinical assessment tool for decision-making behavior in over 10 neuropsychological deficits (30, 32). However, an increasing number of IGT clinical studies have highlighted unresolved issues and invalidations of the tool based on the expected value perspective and the net score of the expected value index (33, 37, 40–42, 45). The present study confirmed that the expected value score [(C+D) − (A+B)] is incapable of distinguishing between the decision-making behaviors of IGD cases and those of controls, which also confirms the findings of other IGT-IA studies (23–27).

### The Gain–Loss Frequency Viewpoint: The Prominent Deck B Phenomenon

The present study found that the mean numbers of cards selected showed a preference for decks B, C, and D rather than deck A, which means that the prominent deck B phenomenon was a feature of both groups. This illustrates the effects of gain–loss frequency and replicates the findings of other studies (33–48). However, although the prominent deck B phenomenon was shown in both groups, there was no difference between the groups in the number of selections of deck B. This implies that all participants were more sensitive to the gain–loss frequency rather than the expected value in the cIGT. To be consistent with Young’s assessment criterion for IA (2, 3), which is based mostly on the DSM diagnostic for substance addiction and pathological gambling, IA cases should be relatively sensitive to the high-frequency gain decks B and D in the IGT. However, the present findings show that the IGD group was no further sensitive to the high-frequency gain decks than the controls (see **Tables 4** and **6**, **Figures 1** and **4**). Consequently, the gain–loss frequency may also be incapable of distinguishing between dynamic decision patterns in the IGT.

### The Learning Curve: Expected Value vs. Gain–Loss Frequency

As **Figure 4** shows, there was no significant difference between the two groups in the learning curves for each deck. Notably, the learning curves of both groups demonstrated a slightly decreased learning tendency in decks A and B, and a slightly increased learning tendency in decks C and D, although there was no statistical difference between the two groups (**Figure 4**). This learning curve data suggest that, after a number of trials, the participants in both groups (IGD vs. controls) might prefer to choose the good decks (C, D) and to avoid the bad decks (A, B). Nevertheless, it has been estimated that participants may need two or three times the number of trials taken in the standard running of the IGT (100 trials) before the foresighted choice pattern can be reached (37).

### The Implications of This Study

The present study has demonstrated that the cIGT, used as a neuropsychological assessment tool, may not be relevant for distinguishing the choice patterns of IGD cases from those of controls. This observation is clearly supported by other IGT-IA studies (23–27). If we assume that the present study substantiates those findings, the result requires some possible explanations and clarification of the issues. The present study found that the clinical IGT is incapable of distinguishing between the choice patterns and those of controls based on the expected value and gain–loss frequency viewpoints. However, Ko et al. (10) have provided some alternative viewpoints: IA cases may be diagnosed mainly by their uncontrolled Internet use behavior, but these behavioral symptoms (addiction to online gaming, for example) might be only an explicit factor (10). Other neuropsychological disorders might be associated with IGD: for instance, attention deficit hyperactivity disorder, impulse control problems, and anxiety (60, 61). Therefore, if the IGD cases possess such a high level of diversity, as identified in Ko et al.’s hypothesis, it is reasonable to assume that the heterogeneity could contribute to the nonsignificant difference between IGD and controls (10).

### The Limitations of This Study

This study might be one of the first to explore IGD by utilizing the cIGT to compare the performances of IA cases and controls. However, the current study has certain limitations that should be noted. Firstly, as mentioned above, there are some differences between the original IGT (11) and the clinical IGT (30). Hence, the findings of the current study might be contaminated by the effects of version differences (62) and a general confounding of the between-group design. Secondly, the sample sizes of the two groups were almost equal in the original experimental design; at a later stage of the study, however, 18 IA cases were diagnosed as remission cases after serial clinical assessments and treatments, 2 datasets were mislabeled, and 1 dataset went missing. Consequently, the sample sizes of the two groups became unbalanced, which might have added a confounding factor and decreased the statistical power in comparing the IGT performance. Thirdly, the participants not only performed the IGT but also other decision-making tasks (e.g., the affective Go/No-go and the cups task); they also received blood tests and were measured for body mass index. Therefore, their IGT performance might have been affected by the other cognitive tasks and the order effect of the tests and investigations. Additionally, some of the participants were fasting before receiving a blood test. This condition might have influenced the levels of their blood sugar and might have also added a new confounding factor.

## Conclusions

Young (2, 3) used substance addiction and pathological gambling criterion to identify and represent cases of IA, while the clinical version of the IGT (30, 32) is claimed to be able to distinguish the choice behaviors of those two cases (substance addiction and pathological gambling) from those of controls. However, the present study has demonstrated that the cIGT, used as a neuropsychological assessment tool, may not be relevant for distinguishing the choice patterns of IGD cases. Firstly, the results showed that the expected value score was incapable of distinguishing between the decision-making behaviors of IGD cases and those of controls, which confirms the findings of other IGT-IA studies (23–27). Additionally, although the learning curve based on the expected value assumption was significant, this index was also unable to distinguish between IGD cases and controls. Secondly, the prominent deck B phenomenon was revealed in both groups and duplicated the gain–loss frequency effect (21, 26) in the cIGT. However, the differences in the numbers of deck B selections were unable to distinguish between the dynamic choice patterns of the two groups, which makes the prominent deck B phenomenon unsuitable as an appropriate index. Thirdly, the gain–loss frequency indices were also unable to distinguish between IGD cases and controls. In summary, the present study did not find effective indices or evidence for the validity of the clinical tool in evaluating IGD cases in the standard administration of the IGT. The lack of differences between IGD/IA cases and controls in the present research and in previous studies suggests two possible explanations. Firstly, the present observation is that decision behavior deficits do not constitute a representative feature of participants who have been diagnosed with IGD/IA. The second is that the cIGT may not be relevant to distinguish the decision-making patterns of IGD/IA cases from those of controls. To conclude, the findings suggest that the results of the clinical IGT should be interpreted carefully when assessing IGD cases.

## Ethics Statement

Each participant provided informed consent before participating in this experiment, and the study followed the guidelines of the Helsinki Declaration. The experimental process was approved by the Institutional Review Board of Kaohsiung Medical University Hospital (KMUH) (IRB No. 990380).

## Author Contributions

CL and YC provided the main concepts and structure of this manuscript. CL and CW provided the data analysis, representation, statistical testing, and most of the interpretation of the results. CL, YC, JS and CK provided the literature review and discussion. CK provided the clinical assessments of the participants in both groups, monitored the data collection process, and provided some valuable discussion to the manuscript. CL, CW, JS, CK, and YC provided some critical refinements in the final revision.

## Conflict of Interest Statement

The authors declare that the research was conducted in the absence of any commercial or financial relationships that could be construed as a potential conflict of interest. 

## Supplementary Material

Supplementary Table 1 The gain–loss structure of original IGT (11) and clinical IGT (30).
