# Supplementary material for: Is the Clinical Version of the Iowa Gambling Task Relevant for Assessing Choice Behavior in Cases of Internet Addiction?
Source: Front Psychiatry. 2019 May 27;10:232. doi: 10.3389/fpsyt.2019.00232 (PMC6545792; doi:10.3389/fpsyt.2019.00232)
Supplement: Supplementary Table 1 — The gain–loss structure of original IGT (11) and clinical IGT (30). [file DataSheet_1.docx]

**Supplementary Table 1**

**The gain-loss structure of original IGT (Bechara et al., 1994) and clinical IGT (Bechara, 2007)**

| Version | **Original IGT** | | | | | | | | | | | | **Clinical IGT** | | | | | | | |
| --- | --- | --- | --- | --- | --- | --- | --- | --- | --- | --- | --- | --- | --- | --- | --- | --- | --- | --- | --- | --- |
| Deck | **A** | | | **B** | | | **C** | | | **D** | | | **A** | | **B** | | **C** | | **D** | |
| Trial | **Gain** | **Loss** | | **Gain** | **Loss** | | **Gain** | **Loss** | | **Gain** | **Loss** | | **Gain** | **Loss** | **Gain** | **Loss** | **Gain** | **Loss** | **Gain** | **Loss** |
| 1 | **100** | **0** | | **100** | **0** | | **50** | **0** | | **50** | **0** | | **100** |  | **100** |  | **50** |  | **50** |  |
| 2 | **100** | **0** | | **100** | **0** | | **50** | **0** | | **50** | **0** | | **120** |  | **80** |  | **60** |  | **40** |  |
| 3 | **100** | **-150** | | **100** | **0** | | **50** | **-50** | | **50** | **0** | | **80** | **-150** | **110** |  | **40** | **-50** | **45** |  |
| 4 | **100** | **0** | | **100** | **0** | | **50** | **0** | | **50** | **0** | | **90** |  | **120** |  | **55** |  | **45** |  |
| 5 | **100** | **-300** | | **100** | **0** | | **50** | **-50** | | **50** | **0** | | **110** | **-300** | **90** |  | **55** | **-50** | **55** |  |
| 6 | **100** | **0** | | **100** | **0** | | **50** | **0** | | **50** | **0** | | **100** |  | **100** |  | **45** |  | **60** |  |
| 7 | **100** | **-200** | | **100** | **0** | | **50** | **-50** | | **50** | **0** | | **80** | **-200** | **90** |  | **50** | **-50** | **40** |  |
| 8 | **100** | **0** | | **100** | **0** | | **50** | **0** | | **50** | **0** | | **120** |  | **120** |  | **45** |  | **55** |  |
| 9 | **100** | **-250** | | **100** | **-1250** | | **50** | **-50** | | **50** | **0** | | **110** | **-250** | **110** | **-1250** | **60** | **-50** | **50** |  |
| 10 | **100** | **-350** | | **100** | **0** | | **50** | **-50** | | **50** | **-250** | | **90** | **-350** | **80** |  | **40** | **-50** | **60** | **-250** |
| **EV** | **1000** | **-1250** | | **1000** | **-1250** | | **500** | **-250** | | **500** | **-250** | | **1000** | **-1250** | **1000** | **-1250** | **500** | **-250** | **500** | **-250** |
| **GLF** | **10** | **5** | | **10** | **1** | | **10** | **5** | | **10** | **1** | | **10** | **5** | **10** | **1** | **10** | **5** | **10** | **1** |
| 11 | **100** |  | | **100** |  | | **50** |  | | **50** |  | | **110** |  | **110** |  | **55** |  | **55** |  |
| 12 | **100** | **-350** | | **100** |  | | **50** | **-25** | | **50** |  | | **130** | **-350** | **100** |  | **55** | **-25** | **40** |  |
| 13 | **100** |  | | **100** |  | | **50** | **-75** | | **50** |  | | **90** |  | **90** |  | **65** | **-75** | **60** |  |
| 14 | **100** | **-250** | | **100** | **-1250** | | **50** |  | | **50** |  | | **100** | **-250** | **130** | **-1500** | **45** |  | **40** |  |
| 15 | **100** | **-200** | | **100** |  | | **50** |  | | **50** |  | | **120** | **-200** | **120** |  | **70** | **-25** | **45** |  |
| 16 | **100** |  | | **100** |  | | **50** |  | | **50** |  | | **110** |  | **130** |  | **40** |  | **55** |  |
| 17 | **100** | **-300** | | **100** |  | | **50** | **-25** | | **50** |  | | **90** | **-300** | **110** |  | **50** | **-25** | **65** |  |
| 18 | **100** | **-150** | | **100** |  | | **50** | **-75** | | **50** |  | | **130** | **-150** | **90** |  | **60** | **-75** | **70** |  |
| 19 | **100** |  | | **100** |  | | **50** |  | | **50** |  | | **120** | **-250** | **100** |  | **70** |  | **50** |  |
| 20 | **100** |  | | **100** |  | | **50** | **-50** | | **50** | **-250** | | **100** |  | **120** |  | **40** | **-50** | **70** | **-275** |
| **EV** | **1000** | **-1250** | | **1000** | **-1250** | | **500** | **-250** | | **500** | **-250** | | **1100** | **-1500** | **1100** | **-1500** | **550** | **-275** | **550** | **-275** |
| **GLF** | **10** | **5** | | **10** | **1** | | **10** | **5** | | **10** | **1** | | **10** | **6** | **10** | **1** | **10** | **6** | **10** | **1** |
| 21 | **100** |  | | **100** | **-1250** | | **50** |  | | **50** |  | | **120** | **-250** | **120** | **-1750** | **60** |  | **60** |  |
| 22 | **100** | **-300** | | **100** |  | | **50** |  | | **50** |  | | **140** | **-300** | **110** |  | **65** | **-25** | **55** |  |
| 23 | **100** |  | | **100** |  | | **50** |  | | **50** |  | | **110** |  | **140** |  | **55** |  | **65** |  |
| 24 | **100** | **-350** | | **100** |  | | **50** | **-50** | | **50** |  | | **110** | **-350** | **130** |  | **80** | **-50** | **80** |  |
| 25 | **100** |  | | **100** |  | | **50** | **-25** | | **50** |  | | **100** |  | **100** |  | **40** | **-25** | **40** |  |
| 26 | **100** | **-200** | | **100** |  | | **50** | **-50** | | **50** |  | | **120** | **-200** | **110** |  | **60** | **-50** | **80** |  |
| 27 | **100** | **-250** | | **100** |  | | **50** |  | | **50** |  | | **130** | **-250** | **120** |  | **55** |  | **40** |  |
| 28 | **100** | **-150** | | **100** |  | | **50** |  | | **50** |  | | **110** | **-150** | **120** |  | **65** | **-25** | **65** |  |
| 29 | **100** |  | | **100** |  | | **50** | **-75** | | **50** | **-250** | | **140** | **-250** | **140** |  | **40** | **-75** | **55** | **-300** |
| 30 | **100** |  | | **100** |  | | **50** | **-50** | | **50** |  | | **120** |  | **110** |  | **80** | **-50** | **60** |  |
| **EV** | **1000** | **-1250** | | **1000** | **-1250** | | **500** | **-250** | | **500** | **-250** | | **1200** | **-1750** | **1200** | **-1750** | **600** | **-300** | **600** | **-300** |
| **GLF** | **10** | **5** | | **10** | **1** | | **10** | **5** | | **10** | **1** | | **10** | **7** | **10** | **1** | **10** | **7** | **10** | **1** |
| 31 | **100** | **-350** | | **100** |  | | **50** |  | | **50** |  | | **130** | **-250** | **130** |  | **65** | **-25** | **65** |  |
| 32 | **100** | **-200** | | **100** | **-1250** | | **50** |  | | **50** |  | | **120** | **-250** | **140** | **-2000** | **75** |  | **75** |  |
| 33 | **100** | **-250** | | **100** |  | | **50** |  | | **50** |  | | **140** | **-150** | **120** |  | **55** | **-25** | **60** |  |
| 34 | **100** |  | | **100** |  | | **50** | **-25** | | **50** |  | | **130** |  | **110** |  | **60** | **-25** | **65** |  |
| 35 | **100** |  | | **100** |  | | **50** | **-25** | | **50** | **-250** | | **110** | **-150** | **130** |  | **70** | **-25** | **75** | **-325** |
| 36 | **100** |  | | **100** |  | | **50** |  | | **50** |  | | **150** | **-300** | **150** |  | **65** |  | **85** |  |
| 37 | **100** | **-150** | | **100** |  | | **50** | **-75** | | **50** |  | | **140** | **-350** | **110** |  | **55** | **-75** | **45** |  |
| 38 | **100** | **-300** | | **100** |  | | **50** |  | | **50** |  | | **120** |  | **150** |  | **75** | **-25** | **55** |  |
| 39 | **100** |  | | **100** |  | | **50** | **-50** | | **50** |  | | **150** | **-350** | **120** |  | **45** | **-50** | **70** |  |
| 40 | **100** |  | | **100** |  | | **50** | **-75** | | **50** |  | | **110** | **-200** | **140** |  | **85** | **-75** | **55** |  |
| **EV** | **1000** | **-1250** | | **1000** | **-1250** | | **500** | **-250** | | **500** | **-250** | | **1300** | **-2000** | **1300** | **-2000** | **650** | **-325** | **650** | **-325** |
| **GLF** | **10** | **5** | | **10** | **1** | | **10** | **5** | | **10** | **1** | | **10** | **8** | **10** | **1** | **10** | **8** | **10** | **1** |
| 41 |  |  | |  |  | |  |  | |  |  | | **140** | **-350** | **140** |  | **70** | **-25** | **70** |  |
| 42 |  |  | |  |  | |  |  | |  |  | | **130** | **-200** | **150** |  | **80** |  | **80** |  |
| 43 |  |  | |  |  | |  |  | |  |  | | **150** | **-250** | **130** |  | **60** | **-25** | **65** |  |
| 44 |  |  | |  |  | |  |  | |  |  | | **140** | **-250** | **120** |  | **65** | **-25** | **70** |  |
| 45 |  |  | |  |  | |  |  | |  |  | | **120** | **-150** | **140** |  | **75** | **-25** | **80** | **-350** |
| 46 |  |  | |  |  | |  |  | |  |  | | **160** |  | **160** | **-2250** | **70** | **-25** | **90** |  |
| 47 |  |  | |  |  | |  |  | |  |  | | **150** | **-150** | **120** |  | **60** | **-75** | **50** |  |
| 48 |  |  | |  |  | |  |  | |  |  | | **130** | **-300** | **160** |  | **80** | **-25** | **60** |  |
| 49 |  |  | |  |  | |  |  | |  |  | | **160** | **-350** | **130** |  | **50** | **-50** | **75** |  |
| 50 |  |  | |  |  | |  |  | |  |  | | **120** | **-250** | **150** |  | **90** | **-75** | **60** |  |
| **EV** |  |  | |  |  | |  |  | |  |  | | **1400** | **-2250** | **1400** | **-2250** | **700** | **-350** | **700** | **-350** |
| **GLF** |  |  | |  |  | |  |  | |  |  | | **10** | **9** | **10** | **1** | **10** | **9** | **10** | **1** |
| 51 |  |  | |  |  | |  |  | |  |  | | **150** | **-350** | **150** |  | **75** | **-25** | **75** |  |
| 52 |  |  | |  |  | |  |  | |  |  | | **140** | **-200** | **160** |  | **85** | **-25** | **85** |  |
| 53 |  |  | |  |  | |  |  | |  |  | | **160** | **-250** | **140** |  | **65** | **-25** | **70** |  |
| 54 |  |  | |  |  | |  |  | |  |  | | **150** | **-250** | **130** |  | **70** | **-25** | **75** |  |
| 55 |  |  | |  |  | |  |  | |  |  | | **130** | **-150** | **150** |  | **80** | **-25** | **85** |  |
| 56 |  |  | |  |  | |  |  | |  |  | | **170** | **-250** | **170** |  | **75** | **-25** | **95** |  |
| 57 |  |  | |  |  | |  |  | |  |  | | **160** | **-150** | **130** |  | **65** | **-75** | **55** |  |
| 58 |  |  | |  |  | |  |  | |  |  | | **140** | **-300** | **170** | **-2500** | **85** | **-25** | **65** | **-375** |
| 59 |  |  | |  |  | |  |  | |  |  | | **170** | **-350** | **140** |  | **55** | **-50** | **80** |  |
| 60 |  |  | |  |  | |  |  | |  |  | | **130** | **-250** | **160** |  | **95** | **-75** | **65** |  |
| **EV** |  | | |  | | |  | | |  | | | **1500** | **-2500** | **1500** | **-2500** | **750** | **-375** | **750** | **-375** |
| **GLF** |  | | |  | | |  | | |  | | | **10** | **10** | **10** | **1** | **10** | **10** | **10** | **1** |
| **Total EV**  **Original IGT**  **(40 trials) vs. Clinical IGT**  **(60 trials)** | **4000** | | **-5000** | **4000** | | **-5000** | **2000** | | **-1000** | **2000** | | **-1000** | **7500** | **-11250** | **7500** | **-11250** | **3750** | **-1875** | **3750** | **-1875** |
| **Total Frequency** | **40** | | **20** | **40** | | **4** | **40** | | **20** | **40** | | **4** | **60** | **45** | **60** | **6** | **60** | **45** | **60** | **6** |
